# Whole genome sequencing data and analyses of the underlying *SUP35* transcriptional regulation for a *Saccharomyces cerevisiae* nonsense suppressor mutant

**DOI:** 10.1016/j.dib.2019.01.042

**Published:** 2019-01-19

**Authors:** Andrew G. Matveenko, Polina B. Drozdova, Svetlana E. Moskalenko, Oleg V. Tarasov, Galina A. Zhouravleva

**Affiliations:** a Department of Genetics and Biotechnology, Saint Petersburg State University, St. Petersburg 199034, Russia; b St. Petersburg Branch, Vavilov Institute of General Genetics, Russian Academy of Sciences, St. Petersburg 199034, Russia; c Laboratory of Amyloid Biology, Saint Petersburg State University, St. Petersburg 199034, Russia

## Abstract

Termination of translation in eukaryotes is governed by two release factors encoded by the *SUP45* and *SUP35* genes in *Saccharomyces cerevisiae*. Previously, a set of mutations in these genes had been obtained. However, the exact sequence change associated with one mutation, *sup35-222*, was not identified by Sanger sequencing of the *SUP35* region. Presented here are whole-genome sequencing data for the *sup35-222* strain, data on copy number variation in its genome along with supporting pulse-field gel electrophoresis experiment data, and the list of single-nucleotide variations that differentiate this strain and its wild-type ancestor. One substitution upstream the *SUP35* gene was located in a sequence corresponding to the Abf1-binding site. Data obtained from the introduction of this variation from *sup35-222* strain into a different wild-type strain, specifically, detection of a nonsense-suppressor phenotype accompanied by a decrease in the Sup35 protein level, are also presented in this article.

**Specifications table**

**Table:** 

Subject area	*Biology*
More specific subject area	*Genetics, genomics, molecular biology (General)*
Type of data	*Tables, images, graphs, figures*
How data was acquired	*WGS was performed with Ion Torrent PGM. Yeast strain construction and analysis was performed using standard techniques.*
Data format	*Raw data*
Experimental factors	*Saccharomyces cerevisiae strains 222-1B-D1606, U-P*^*S*^*-A-GT671, and U-1-A-GT671*
Experimental features	*Whole genome sequencing of the 222-1B-D1606 strain with Ion Torrent PGM. Pulse-field Gel Electrophoresis of the DNA extracted from the 222-1B-D1606 strain. Analysis of reference genome coverage and detection of single-nucleotide variations from the obtained reads.* In silico *search for transcription factor-binding sites in the* SUP35 *promoter. Construction and phenotypic analysis of novel* S. cerevisiae *strain with mutant* sup35-222 *promoter.*
Data source location	*Dpt. of Genetics and Biotechnology, Saint-Petersburg State University, St. Petersburg, Russia*
Data accessibility	*Raw sequencing data is available from the NCBI SRA database under the accession number at*https://www.ncbi.nlm.nih.gov/sra/SRX1484451*. Processed data are available from a public repository*https://github.com/drozdovapb/PeterhofYeastHub*(single-nucleotide variations are available at*https://github.com/drozdovapb/PeterhofYeastHub/blob/master/sacCer3/222.vcf.gz*, while the* de novo *assembled contigs can be accessed at*https://github.com/drozdovapb/PeterhofYeastHub/blob/master/222_contigs.2bit*). Tracks with SNVs in 222-1B-D1606 relative to the reference* S. cerevisiae *genome are available from UCSC genome browser within the Peterhof_yeasts track hub at*http://genome.ucsc.edu/cgi-bin/hgTracks?db=sacCer3&lastVirtModeType=default&lastVirtModeExtraState=&virtModeType=default&virtMode=0&nonVirtPosition=&position=chrIV%3A806387%2D811017&hgsid=700542643_mBI1kiL5N4AXLNzv4RcMwv26jmmt. *Other data are available with this article.*
Related research article	Drozdova, P.B., Tarasov, O.V., Matveenko, A.G., Radchenko, E.A., Sopova, J.V., Polev, D.E., Inge-Vechtomov, S.G., Dobrynin, P.V., 2016. Genome sequencing and comparative analysis of Saccharomyces cerevisiae strains of the Peterhof genetic collection. PLoS One. 11, e0154722. https://doi.org/10.1371/journal.pone.0154722

**Value of the data**

• Data on the role of Abf1-dependent transcriptional regulation in translational readthrough is useful for uncovering new mechanisms of translational fidelity control.
• Data on the whole genome sequencing of *S. cerevisiae* strain with nonsense-suppressor phenotype can be useful for the studies of translation, specifically, identification of mutations that accumulate in cells with high level of translational readthrough.
• Genome coverage data, which include the duplication of the region containing *SUP35*, can be used in studies of chromosomal rearrangements and their adaptive role.
• The mutation in the *SUP35* promoter identified here is valuable for studies of transcription of the release factor genes as well as for the mechanisms of Abf1-derived regulation of transcription.
• The data on the phenotypic analysis of strains with substitutions in the *SUP35* promoter can be useful for studies of [*PSI*^+^] prion and its maintenance during a decrease in the Sup35 protein level. It also can be used for the studies of termination of translation.

## 1 Data

**Fig. 1:**
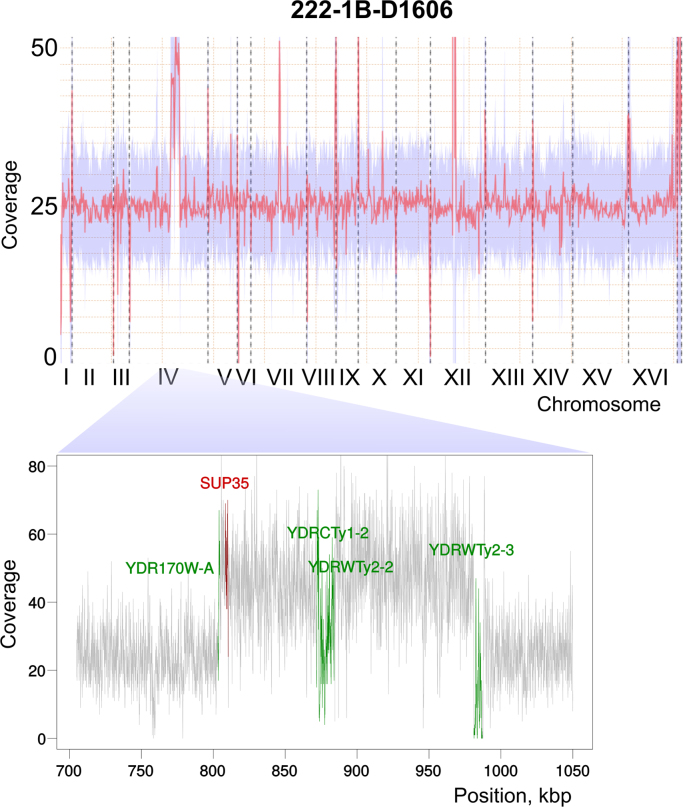
Data on copy number variation in the 222-1B-D1606 genome. Shown is the graphic representation of the data on coverage estimation throughout the 222-1B-D1606 genome. The amplified region on the chromosome XII corresponds to ribosomal DNA repeats. The presumably duplicated region of the chromosome IV is magnified. Positions of the transposon-derived sequences within this region are highlighted in green.

**Fig. 2:**
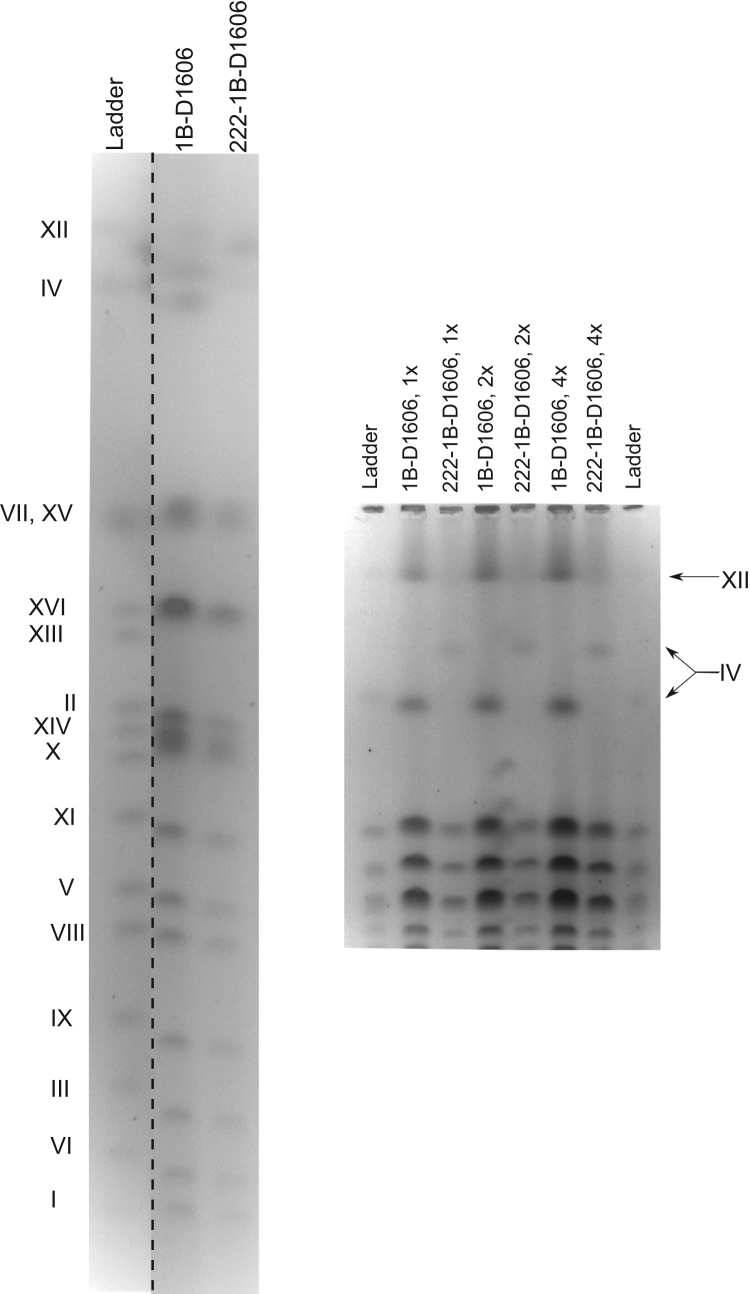
Analysis of the chromosome IV length in 222-1B-D1606 with PFGE. The data on the electrophoretic separation of yeast chromosomes in 1% agarose gel with subsequent ethidium bromide staining are shown. The commercial PFGE standard (Bio-Rad) was used as a ladder. Lanes at the right panel differ by the amount of material loaded into the corresponding well (indicated by 1×, 2× and 4×).

**Fig. 3:**
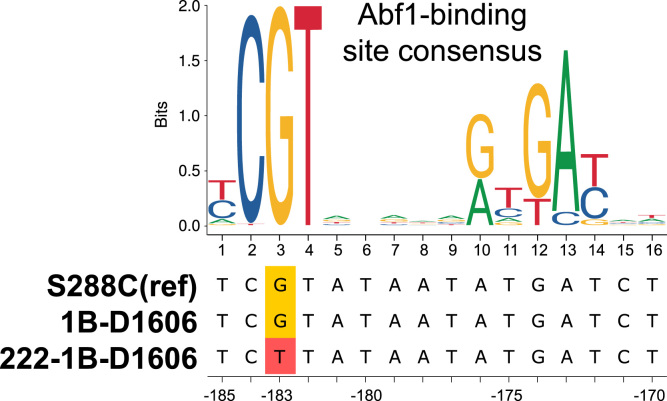
Alignment of *SUP35* promoter sequences from 222-1B-D1606, its ancestor, and the reference strain (S288C).

**Fig. 4:**
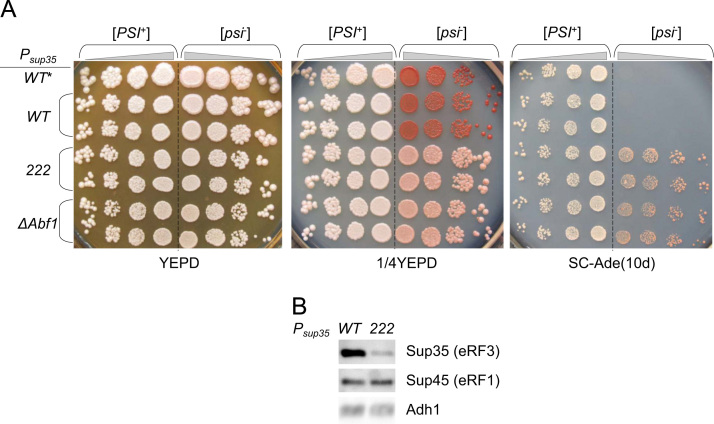
The data on the effects of the *P*_*sup35-222*_ and *P*_*sup35ΔAbf1*_ promoter variants on nonsense suppression and eRF3 abundance. A. *SUP35* regulated by the wild-type promoter_*,*_ in the initial strain (WT*) was replaced with *SUP35* under control of either wild-type (*WT*, as a control) or mutant (*222* or *ΔAbf1*) promoter. Shown are tenfold serial dilutions of representative clones spotted onto full (YEPD, 1/4YEPD) or selective (SC-Ade) media after 4 or 10 days of incubation, respectively. [*PSI*^+^] and [*psi*^−^] are U-P^S^-A-GT671 and U-1-A-GT671 strains, respectively. B. Sup35 (eRF3) protein level in wild-type and mutant strains. Proteins were extracted from clones with *sup35-222* or wild-type promoter variants and processed as described in Protein analysis section.

Raw whole-genome sequencing data for the *sup35-222 Saccharomyces cerevisiae* mutant strain was produced with Ion Torrent PGM. The genome was then assembled with the obtained reads (assembly statistics are present in Table 1 and Table S1). Genome coverage analysis performed by aligning short reads to the reference S288C genome showed a duplicated region of the chromosome IV that included the *SUP35* ORF (Fig. 1). Pulse field gel electrophoresis (PFGE) analysis was then performed to compare chromosome lengths in the mutant strain and its ancestor and thus find if the duplication was located on the same chromosome (Fig. 2). As the ancestor strain, 1B-D1606, had been sequenced previously, single nucleotide variations (SNVs) between 1B-D1606, 222-1B-D1606 and reference strain were compared (Table S2). One single-nucleotide variation upstream the *SUP35* coding sequence was identified. This substitution destroys potential Abf1-binding site in the *SUP35* promoter (Fig. 3). Introducing the same variation, as well as deletion of the Abf1-binding site, in the *SUP35* promoter into another strain led to detection of nonsense suppressor phenotype (Fig. 4A), accompanied by a decrease in the Sup35 protein level (Fig. 4B).

**Table 1:** Statistics of 222-1B-D1606 and 1B-D1606 *de novo* genome assemblies.

***Genome statistics***	***222-1B-D1606***	***1B-D1606***
Number of contigs (>500 bp)	854	480
Reference genome fraction (%)	94.12	94.157
Duplication ratio	1.013	1.006
Number of genes found	5771 + 441 part	6010 + 203 part
Largest alignment	139,569	165,926
Total aligned length, bp	11,559,970	11,499,891
N50	32,468	72,884
Reference	This work	[3]

## 2 Experimental design, materials, and methods

### 2.1 Sequencing and *in silico* analysis

The 1B-D1606 and 222-1B-D1606 strains were obtained and described previously [1], [2]. Whole-genome sequencing was conducted with Ion Torrent PGM. Next generation sequencing and data analysis were performed as described earlier [3] with minor modifications. Genomic DNA was prepared with mechanical disruption of yeast cells [4]. SPAdes [5] and Quast [6] were used for the *de novo* genome assembly and estimation of assembly quality, respectively. Bowtie [7], qualimap [8], and UGENE [9], [10] were used to generate and analyse alignment of reads with the reference genome. The ggplot2 package [11] for R [12] was also used for coverage visualization. Samtools [13], vcftools v1.0 [14] and snpEff v4.1 [15] were used for SNV calling and annotation. Positions with low quality (*q* < 30) and low coverage (DP < 3), as well as heterozygous indels and variations in the repeat regions, were filtered out. The difference between the two strains by SNVs relative to the S288C genome was assessed with bedtools-intersect [16]. Complete genome sequence of 1B-D1606 and its assembly statistics have been published previously [3]. Raw data for the 222-1B-D1606 genome sequencing are available from the NCBI SRA database (https://www.ncbi.nlm.nih.gov/sra/SRX1484451), and the SNVs corresponding to 1B-D1606 and 222-1B-D1606 are available from UCSC genome browser (http://genome.ucsc.edu/cgi-bin/hgHubConnect#publicHubs under Peterhof_yeast_hub). The data on single nucleotide variations, as well as the *de novo* genome assembly of the 222-1B-D1606 strain, are also available at https://github.com/drozdovapb/PeterhofYeastHub (https://github.com/drozdovapb/PeterhofYeastHub/blob/master/sacCer3/222.vcf.gz and https://github.com/drozdovapb/PeterhofYeastHub/blob/master/222_contigs.2bit for SNV data and contigs, respectively).

Search for transcription factors that bind *SUP35* promoter variants was carried out using oPOSSUM3.0 (http://opossum.cisreg.ca/oPOSSUM3). Consensus for the Abf1-binding site was downloaded from the JASPAR2018 database ([17]; http://jaspar.genereg.net/matrix/MA0265.1).

The mutation in the *SUP35* promoter in 222-1B-D1606 was confirmed by Sanger sequencing of the PCR fragment amplified from genomic DNA of the strain with primers sup35_f (5′-CCAACCCTACGGTAGAAAA-3′) and SUP35_R (5′-GGATTGAATTGCTGCTGATAAC-3′). Sanger sequencing was performed with ABI Prism 3500xl. All sequencing reactions were performed at the Research Resource Center “Molecular and Cell Technologies” of the Saint Petersburg State University.

### 2.2 Pulse field gel electrophoresis

Genomic DNA extraction for pulse field gel electrophoresis (PFGE) was performed in low melting point agarose according to the described method [18]. PFGE was run with the CHEF Mapper XA pulse field electrophoresis system (Bio-Rad) according to the manufacturer׳s recommendations.

### 2.3 Yeast plasmid and strain construction

Standard media and yeast cultivation techniques were used [19]. The pRSU1 and pRSU2 plasmids [20] were used for expression of the *SUP35* gene under its wild-type promoter. For expression of *SUP35* under control of the mutant promoter, the pRSU1-222 plasmid was constructed by cloning PstI-MluI-restricted PCR fragment, which was obtained by amplifying the *SUP35* 5′-UTR and a part of ORF from 222-1B-D1606 genomic DNA, into the same sites of pRSU1, thus substituting the wild-type promoter sequence with the mutant one. Correct fragment insertion was confirmed by Sanger sequencing of the plasmid. *SUP35* regulated by its promoter with deletion of Abf1-binding site was introduced on the pNR-ΔABF1 plasmid [21]. The pRSU2 plasmid was substituted with pRSU1, pRSU1-222, or pNR-ΔABF1 via plasmid shuffling on 5-FOA medium [19] in the U-P^S^-A-GT671, or U-1-A-GT671 strains. These strains are derivatives of the GT671 strain (*MATα ade1-14 his3 lys2 ura3-2 leu3,112 trp1 sup35::HIS3* [*CEN LEU2 SUP35*] [*psi*^-^][*pin*^-^]), which is a kind gift from Y.O. Chernoff. U-P^S^-A-GT671 strain was selected after substituting *SUP35*-bearing plasmid for pRSU2 in L-P-A-GT671, a *MAT*a segregant which was obtained after mating GT671 with GT81-1C strain [22]. U-1-A-GT671 was selected in U-P-A-GT671 progeny as a clone that had spontaneously lost the [*PSI*^+^] prion.

### 2.4 Protein analysis

Protein extraction was performed using the modified alkaline lysis method [23]. SDS-PAGE, semi-dry transfer onto PVDF membrane and western blot were carried out using standard techniques [24]. Antibodies SE4291 [2], SE-45-2 [25] and ADH1A (LsBio, #LS-C68862) were used to detect Sup35, Sup45, and Adh1, respectively. ECL Select Western Blotting Detection Reagent (Amersham) was used for antibody detection, and images were acquired with GeneGnome (Syngene) hardware and software.

## Appendix A Supplementary material

Supplementary Table S1. Detailed statistics of 222-1B-D1606 and 1B-D1606 *de novo* genome assemblies.

.

Supplementary Table S2. List of SNVs found in 222-1B-D1606 and 1B-D1606 compared to reference genome (S288C) in VCF v4.1 format.

## References

[1] Moskalenko S., Chabelskaya S., Inge-Vechtomov S., Philippe M., Zhouravleva G. (2003). Viable nonsense mutants for the essential gene SUP45 of *Saccharomyces cerevisiae*. BMC Mol. Biol..

[2] Chabelskaya S., Kiktev D., Inge-Vechtomov S., Philippe M., Zhouravleva G. (2004). Nonsense mutations in the essential gene SUP35 of *Saccharomyces cerevisiae* are non-lethal. Mol. Genet. Genom..

[3] Drozdova P.B., Tarasov O.V., Matveenko A.G., Radchenko E.A., Sopova J.V., Polev D.E., Inge-Vechtomov S.G., Dobrynin P.V. (2016). Genome sequencing and comparative analysis of *Saccharomyces cerevisiae* strains of the Peterhof genetic collection. PLoS One.

[4] Lada A.G., Stepchenkova E.I., Waisertreiger I.S., Noskov V.N., Dhar A., Eudy J.D., Boissy R.J., Hirano M., Rogozin I.B., Pavlov Y.I. (2013). Genome-wide mutation avalanches induced in diploid yeast cells by a base analog or an APOBEC deaminase. PLoS Genet..

[5] Bankevich A., Nurk S., Antipov D., Gurevich A.A., Dvorkin M., Kulikov A.S., Lesin V.M., Nikolenko S.I., Pham S., Prjibelski A.D., Pyshkin A.V. (2012). SPAdes: a new genome assembly algorithm and its applications to single-cell sequencing. J. Comput. Biol..

[6] Gurevich A., Saveliev V., Vyahhi N., Tesler G. (2013). QUAST: quality assessment tool for genome assemblies. Bioinformatics.

[7] Langmead B., Salzberg S.L. (2012). Fast gapped-read alignment with Bowtie 2. Nat. Methods.

[8] Okonechnikov K., Conesa A., García-Alcalde F. (2015). Qualimap 2: advanced multi-sample quality control for high-throughput sequencing data. Bioinformatics.

[9] Okonechnikov K., Golosova O., Fursov M. (2012). UGENE team. Unipro UGENE: a unified bioinformatics toolkit. Bioinformatics.

[10] Golosova O., Henderson R., Vaskin Y., Gabrielian A., Grekhov G., Nagarajan V., Oler A.J., Quiñones M., Hurt D., Fursov M., Huyen Y. (2014). Unipro UGENE NGS pipelines and components for variant calling, RNA-seq and ChIP-seq data analyses. PeerJ..

[11] Wickham H. (2009). ggplot2: Elegant Graphics for Data Analysis.

[12] R Core Team (2017). R: A Language and Environment for Statistical Computing.

[13] Li H., Handsaker B., Wysoker A., Fennell T., Ruan J., Homer N. (2009). The sequence alignment/map format and SAMtools. Bioinformatics.

[14] Danecek P., Auton A., Abecasis G., Albers C.A., Banks E., DePristo M.A. (2011). The variant call format and VCFtools. Bioinformatics.

[15] Cingolani P., Platts A., Wang L.L., Coon M., Nguyen T., Wang L., Land S.J., Lu X., Ruden D.M. (2012). A program for annotating and predicting the effects of single nucleotide polymorphisms, SnpEff: snps in the genome of Drosophila melanogaster strain w1118; iso-2; iso-3. Fly.

[16] Quinlan A.R., Hall I.M. (2010). BEDTools: a flexible suite of utilities for comparing genomic features. Bioinformatics.

[17] Mathelier A., Fornes O., Arenillas D.J., Chen C.Y., Denay G., Lee J. (2015). JASPAR 2016: a major expansion and update of the open-access database of transcription factor binding profiles. Nucleic Acids Res..

[18] Zhang H., Zeidler A.F., Song W., Puccia C.M., Malc E., Greenwell P.W. (2013). Gene copy-number variation in haploid and diploid strains of the yeast *Saccharomyces cerevisiae*. Genetics.

[19] Kaiser C., Michaelis S., Mitchell A. (1994). Methods in Yeast Genetics: A Cold Spring Harbor Laboratory Course Manual.

[20] Volkov K.V., Aksenova A.Y., Soom M.J., Osipov K.V., Svitin A.V., Kurischko C. (2002). Novel non-Mendelian determinant involved in the control of translation accuracy in *Saccharomyces cerevisiae*. Genetics.

[21] Ryabinkova N.A., Borchsenius A.S., Inge-Vechtomov S.G. (2009). The influence of mutations at ATG triplets of the open reading frame SUP35 on viability of the yeast *Saccharomyces cerevisiae*. Russ. J. Genet..

[22] Chernoff Y.O., Newnam G.P., Kumar J., Allen K., Zink A.D. (1999). Evidence for a protein mutator in yeast: role of the Hsp70-related chaperone ssb in formation, stability, and toxicity of the [PSI] prion. Mol. Cell. Biol..

[23] Zhang T., Lei J., Yang H., Xu K., Wang R., Zhang Z. (2011). An improved method for whole protein extraction from yeast *Saccharomyces cerevisiae*. Yeast.

[24] Sambrook J., Fritsch E.F., Maniatis T. (1989). Molecular Cloning: A Laboratory Manual.

[25] Kiktev D., Moskalenko S., Murina O., Baudin-Baillieu A., Rousset J.P., Zhouravleva G. (2009). The paradox of viable sup45 STOP mutations: a necessary equilibrium between translational readthrough, activity and stability of the protein. Mol. Genet. Genom..

